# Hyponatremia as a prognostic and predictive factor in metastatic renal cell carcinoma

**DOI:** 10.1038/sj.bjc.6605563

**Published:** 2010-02-09

**Authors:** A N Jeppesen, H K Jensen, F Donskov, N Marcussen, H von der Maase

**Affiliations:** 1 Department of Oncology, Aarhus University Hospital, Nørrebrogade 44, 8000 Aarhus C, Denmark; 2 Department of Experimental Clinical Oncology, Aarhus University Hospital, Nørrebrogade 44, 8000 Aarhus C, Denmark; 3 Department of Clinical Pathology, Odense University Hospital, Winsløewparken 15, 5000 Odense C, Denmark; 4 Department of Oncology, Rigshospitalet, Copenhagen University Hospital, Blegdamsvej 9, 2100 København Ø, Denmark

**Keywords:** renal cell carcinoma, sodium, metastatic, mortality, predictive

## Abstract

**Background:**

Low serum sodium has recently been associated with poor survival in *localised* renal cell carcinoma (RCC). We now show the prognostic effect of serum sodium in patients with *metastatic* RCC (mRCC).

**Methods:**

Cohort A comprised 120 consecutive patients with mRCC receiving subcutaneous, low-dose interleukin-2 and interferon-*α*. Hyponatremia was assessed in univariate and multivariate analyses. An independent cohort of another 120 patients with mRCC was used for validation (cohort B).

**Results:**

In cohort A, estimated 5-year survival was 15% and median survival was 15.1 months. Serum sodium ranged between 126 and 144 mM. Twenty-four patients (20%) had serum sodium levels below normal range (<136 mM). In multivariate analysis, significant independent risk factors for short survival were low serum sodium (*P*=0.014), high neutrophils (*P*=0.018), lactate dehydrogenase >1.5 upper normal level (*P*=0.002), and number of metastatic sites (+3) (*P*=0.003). In cohort B, serum sodium ranged between 128 and 146 mM. Seventeen patients (14%) had sodium levels below normal range. In multivariate analysis, serum sodium was validated as an independent prognostic factor (*P*=0.001). A significant association between lack of response and hyponatremia was observed in both cohorts (*P*=0.003 and *P*=0.02, respectively).

**Conclusion:**

Low serum sodium is a new, validated, independent prognostic, and predictive factor in patients with mRCC.

Renal cell carcinoma (RCC) represents approximately 2% of all cancers worldwide (Parkin *et al*, 2005). Approximately, half of the patients will suffer from metastatic disease, and untreated, the 5-year survival rate for these patients is <2% (Cohen and McGovern, 2005). Selection of patients to optimal treatment are based on prognostic models incorporating patient and disease related clinical prognostic factors (Choueiri, 2009, pp 154–166) with the Memorial Sloan Kettering Cancer Center (MSKCC) prognostic model as the most extensively used (Motzer *et al*, 2002). However, further examination of potentially new predictive and prognostic markers is highly warranted.

Serum sodium is a readily available, easily obtained and routinely measured plasma electrolyte. However, low serum sodium, hyponatremia, is an often underdiagnosed and untreated electrolyte disturbance. Until recently, no predictive or prognostic role of low serum sodium has been recognised. This is despite hyponatremia is the most common electrolyte disorder in hospitalised patients (Asadollahi *et al*, 2007; Patel and Balk, 2007; Ghali, 2008). However, hyponatremia is associated with poor outcome in several medical conditions, such as liver cirrhosis (Luca *et al*, 2007; Kim *et al*, 2008), congestive heart failure (Gheorghiade *et al*, 2007; Rossi *et al*, 2007; Gotsman *et al*, 2008; Rusinaru *et al*, 2009), and infectious diseases as pneumonia (Nair *et al*, 2007), childhood meningitis (Chao *et al*, 2008), and necrotising soft-tissue infection (Yaghoubian *et al*, 2007). Moreover, hyponatremia has recently been associated with poor overall survival in hepatocellular carcinoma (Huo *et al*, 2008), gastric cancer (Kim *et al*, 2007), and small cell lung cancer (Gandhi and Johnson, 2006). In *localised* RCC, serum sodium level below median values has recently been associated with poor disease free and overall survival after nephrectomy (Vasudev *et al*, 2008). Hyponatremia may thus be an indicator of adverse prognosis in non-malignant as well as malignant diseases.

The objective of this study was to evaluate the predictive and prognostic role of hyponatremia in patients with metastatic RCC (mRCC) in two consecutive groups of patients.

## Materials and methods

### Patients

A cohort of 123 consecutive patients was treated with low-dose, subcutaneous interleukin-2 (IL-2) and interferon (IFN)-*α* from September 2002 to August 2004 at the Department of Oncology, University Hospital of Aarhus, Denmark (cohort A). Three patients were excluded as the treatment represented re-induction with IL-2/IFN after late progression of disease (*N*=2) and because of lack of confirmation of RCC at central review (*N*=1), thus leaving 120 patients eligible for this study. Patients assigned for treatment had histologically confirmed RCC, progressive metastatic disease, ECOG performance status ⩽2, age 18–70 years, adequate organ function, and no brain metastases. Objective response was assessed according to RECIST (Therasse *et al*, 2000). Stable disease was defined as no progression at 6 months after initiation of treatment. Overall survival time was defined as time from initiation of treatment to time of death or last follow-up. Survival status was updated on 24 August 2008. Clinical baseline data were retrospectively obtained through chart review. The presence of comorbidity was retrospectively registered for all patients. Concomitant medication was inadequately registered in the patient records and was therefore not scored. No patients were lost to follow-up. The local ethical committee approved the study.

For the assessment of histology subtype, tumour samples were collected from the routine pathologic evaluation obtained at primary diagnosis or at verification of recurrence and reviewed centrally by a single senior pathologist (NM) according to UICC (Storkel *et al*, 1997). Three patients could not be classified because of insufficient tumour material and insufficient initial pathology report, and were thus categorised as unclassified.

A second, independent cohort was used for validation of the results. The validation-cohort consisted of 120 patients with mRCC from the same institution (cohort B). These patients were treated in different phase II trials with low or intermediate dose subcutaneous IL-2 with or without IFN-*α* and histamine dihydrochloride from February 1999 to August 2002. Response rates were defined according to WHO (Miller *et al*, 1981). Survival status was updated on 30 November 2008. Data were prospectively collected. The histological subtype in this cohort was also centrally reviewed (NM). Clinical results of this patient group have been published earlier (Donskov and von der Maase, 2006).

### Treatment

Patients in cohort A were treated in an outpatient setting, consisting of one priming-week of daily IFN-*α* followed by up to six treatment cycles of 4 weeks with IFN*α*-2b (Introna, Schering-Plough, Denmark, 3 MIU as a fixed dose s.c. once daily 5 days per week throughout the treatment) and IL-2 (Aldesleukin, rIL-2, Proleukin, Chiron, The Netherlands, 2.4 MIU m^–2^, s.c. two times daily 5 days per week, weeks 1 and 2 every cycle). Assessment for objective response was carried out every three cycles. Treatment was discontinued in case of progressive disease or unacceptable toxicity.

In all, 40 patients had second-line treatment after progression with IL-2 and 15 had more than one treatment. Four patients were re-induced with IL-2, 22 were included in a randomised phase III trial with lapatinib *vs*. medroxyprogesteron, 12 were included in a phase I/II study with dendritic cell vaccination and 2 patients were included in a study with IL-21. Additional 14 patients were treated with sorafenib and 5 patients with sunitinib.

### Analysis of serum sodium

Serum sodium was performed as a part of the routine laboratory assessment before treatment. Serum sodium was analysed using a certified procedure ‘Cobas Integra 700’ until June 2004 and ‘Cobas Integra 800’ from July 2004 (Palmer *et al*, 1995; Redondo *et al*, 2003).

### Statistical methods

Uni- and multivariate survival analyses were based on Cox proportional hazards regressions model. The univariate parameters with *P*<0.05 were used in the multivariate analyses using backwards selection. The variables were tested for interaction and the assumption for proportional hazards was verified. All calculations were performed according to intention to treat. All tests were two-sided, and *P*<0.05 was considered statistically significant.

For the survival analysis, the variables were dichotomised. Clinical factors: median age, >58 years *vs* ⩽58 years; ECOG performance status, ⩾2 *vs* 0.1; weight loss, >10 *vs* ⩽10%, metastases-free interval, <1 *vs* ⩾1 year; number of disease sites, ⩾3 *vs* 1.2, and sites of metastases (lung, bone, liver, primary tumour *in situ*, adrenal gland, and lymph nodes) Biochemical factors: serum sodium, below normal *vs* normal; haemoglobin, below normal *vs* normal; leukocytes, above normal *vs* normal; neutrophils, above normal *vs* normal; platelets, above normal *vs* normal, plasma albumin, below normal *vs* normal; plasma calcium at pH=7.4, above normal *vs* normal; alkaline phosphatase, above normal *vs* normal; lactate dehydrogenase, ⩾1.5 upper normal limit (UNL) *vs* <1.5 UNL; bilirubin, above normal *vs* normal; alanine aminotransferase (ALAT), above normal *vs* normal.

Primary tumour *in situ* was not included in the multivariate analysis because of close interaction with metastasis-free interval. The dichotomised value of serum sodium was correlated with the clinical and biochemical variables using Fisher's exact test. Statistical analyses were performed using Stata version 9.1 (Stata Corp., 2003, College Station, TX, USA)

## Results

### Clinical results

**Figure 1:**
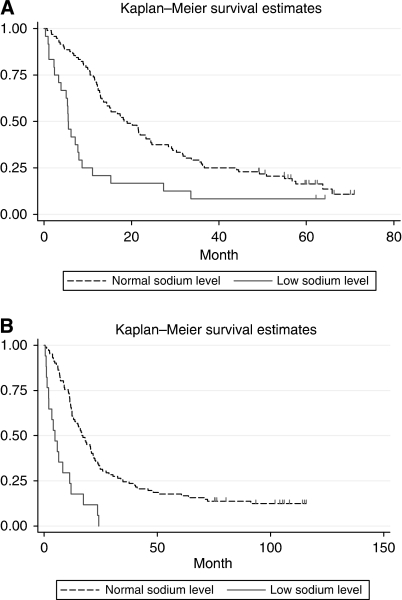
(**A**) Survival and baseline serum sodium level, cohort A (*N*=120). (**B**) Survival and baseline serum sodium level, cohort B (*N*=119).

Baseline characteristics for cohort A are shown in Table 1. The median age was 58.3 years and 68% were men. The median survival time was 15.1 month (range: 0.3–71.0+), and 103 patients had died by the end of follow-up. The estimated 5-year survival rate was 15%, (Figure 1A). Of the 120 patients, 111 had clear cell carcinoma, 4 papillary, 2 chromofobe, and 3 had unclassified tumours.

On the basis of intention to treat, the overall response rate was 14%, including 3 patients with a complete response (3%) and 14 patients with a partial response (12%). Thirty-six patients (30%) had stable disease for a minimum of 6 months and 47 (39%) had progressive disease. Twenty patients (17%) were not evaluable for response because of early treatment termination due to toxicity. Fourteen patients (12%) had curative intended surgery of residual disease after completed immunotherapy. A total of seven patients (6%) had no evidence of disease at 49.1–71+ months after treatment.

### Serum sodium

In cohort A, serum sodium values ranged between 126 and 144 mM (mmol l^–1^, median 138 mM). Twenty-four patients (20%) had sodium below the normal range (<136 mM). Hyponatremia was significantly associated with the following variables: performance status (2+), number of sites (3+), primary tumour *in situ*, weight loss >10%, and MSKCC poor risk group. Hyponatremia was also significantly associated with the following biochemical variables: leukocytes above normal, albumin below normal, alkaline phosphatase above normal, and low haemoglobin (Table 2a). Assessing patients with clear cell histology only (*N*=111), similar associations were observed, however, primary tumour *in situ* was only marginally significant (*P*=0.06).

Twenty patients (83%) with hyponatremia received three or less series of IL-2. Nine of these discontinued early because of toxicity and were as such not evaluable for response, whereas 11 had progressive disease at first evaluation (after three treatment cycles). In contrast, 51 (53%) patients with normal sodium levels received more than three series of IL-2, because of response to treatment or stable disease allowing for continuation of treatment beyond the first evaluation (Table 2b).

In general, comorbidity was infrequent and mild. Among the 24 patients with hyponatremia, 5 (21%) had hypertension, 1 (4%) had sequelae from a cerebral insult, and 4 (17%) had diabetes mellitus. For the 96 patients with normal level of natrium, 20 (21%) had hypertension, 2 (2%) had previously had a cerebral insult, 3 (3%) had previously had a myocardial infarction, 5 (5%) had diabetes mellitus, and 4 (4%) had asthma or COPD.

### Univariate survival analyses

Clinical factors significantly associated with short survival in cohort A were: performance status (2+), metastasis-free interval (<1 year), number of metastatic sites (3+), presence of bone-metastases, primary tumour *in situ*, low serum sodium, blood neutrophiles (>normal), serum albumin (<normal), and lactate dehydrogenase (>1.5 UNL) (Table 3).

Patients with hyponatremia before treatment (*N*=24) had a median overall survival of only 5.5 months (range 0.3–64.1 months), whereas patients with normal sodium values at baseline (*N*=96) had a median survival of 18.6 months (range 0.7–71.0 months) (*P*<0.001) (Figure 1A). Two patients with low serum sodium had a survival above 40 months. Both these patients had a baseline serum sodium level of 134 mM, just below normal value.

When analysing clear cell tumours only (*N*=111), the identical nine parameters were significantly associated with poor overall survival.

### Multivariate survival analysis

For cohort A, significant baseline parameters from the univariate analyses were included in the multivariate analysis. Significant independent risk factors for short survival were low serum sodium (HR 1.90, CI 1.1–3.2, *P*=0.014), neutrophils above normal (HR 1.75, CI 1.1–2.8, *P*=0.018), lactate dehydrogenase >1.5 ULN (HR 2.09, CI 1.3–3.3, *P*=0.002), and number of metastatic sites (+3) (HR 1.92; CI 1.3–2.9, *P*=0.003) (Table 4a). When analysing patients with clear cell carcinomas only (*N*=111), independent factors for poor survival were performance status (2+), metastasis-free interval (<1 year), serum albumin (<normal), and lactate dehydrogenase (>1.5 ULN).

### Validation of baseline serum sodium as a prognostic factor

In the independent cohort (cohort B; *N*=120), the range of serum sodium was 128–148 mM with a median value of 140 mM. Patients with baseline hyponatremia (*N*=17) had a median overall survival of only 4.8 months (range 0.5–24.2 months), whereas patients with normal sodium values at baseline (*N*=102) had a median survival of 16.9 months (range 0.5–115.8 months) (*P*<0.001) (Figure 1B). All long-term survivors had normal serum sodium level. In this cohort B, low serum sodium was validated as an independent prognostic factor for survival in the multivariate analysis (HR 2.98, CI 1.54–5.77, *P*=0.001) (Table 4b). Assessing clear cell carcinomas only in this cohort, serum sodium was also an independent prognostic factor for survival in the multivariate analysis (HR 3.52, CI 1.85–6.69, *P*=0.000).

### Sodium as a predictive factor

In both cohort A and B, hyponatremia was associated with lack of response. In cohort A, 15 responders (88%), including all with a complete response, had normal baseline serum sodium levels. In cohort B, 13 responders (87%) had normal serum sodium. A significant association between lack of response and low serum sodium was observed in both cohorts (*P*=0.003 and *P*=0.02, respectively).

## Discussion

This study is to our knowledge the first to show and validate low baseline serum sodium as a prognostic factor for short survival and a predictive factor for lack of response in patients with mRCC receiving IL-2-based therapy. Serum sodium is a readily available, easily obtained and routinely measured plasma electrolyte. Thus, hyponatremia may have the potential to be easily incorporated into prognostic models optimising outcome prediction. Moreover, patients with hyponatremia should have more intensive cancer care as this patient group represents a subgroup with a dismal prognosis with a median survival of only approximately 5 months.

Serum sodium has remained neglected as a significant clinical feature despite being one of the most frequently obtained blood tests in daily clinical practise. Most often, hyponatremia has been underdiagnosed and untreated by medical staff. This is despite hyponatremia is the most common electrolyte disorder in hospitalised patients (Asadollahi *et al*, 2007; Patel and Balk, 2007). However, within the last few years, a large body of evidence has emerged rendering hyponatremia as a strong general danger signal in many disorders as liver cirrhosis (Luca *et al*, 2007; Kim *et al*, 2008), congestive heart failure (Gheorghiade *et al*, 2007; Rossi *et al*, 2007; Ghali, 2008; Gotsman *et al*, 2008; Rusinaru *et al*, 2009), infections (Nair *et al*, 2007; Yaghoubian *et al*, 2007; Chao *et al*, 2008), and several cancers (Gandhi and Johnson, 2006; Kim *et al*, 2007; Huo *et al*, 2008; Kacprowicz and Lloyd, 2009). Thus, hyponatremia may in fact be an important universal danger signal and an indicator of poor prognosis. Therefore, the association in *localised* RCC between hyponatremia and short disease free and overall survival after nephrectomy (Vasudev *et al*, 2008) is in line with these observations. Similarly, our findings in this study of hyponatremia as an independent, validated predictive and prognostic factor in mRCC just further emphasises and extends the importance of hyponatremia as a significant risk feature.

The reason for hyponatremia in mRCC is largely unknown. In both our cohorts presented in this study, the serum sodium below normal range was only present in a minority of the patients (20% and 14%, respectively). Strikingly, low values of serum sodium were significantly associated with several clinical and biochemical factors known to be associated with poor outcome, among others poor performance status, weight loss, low haemoglobin, and MSKCC poor risk grouping. Importantly, patients with hyponatremia did not have a higher frequency of comorbidity than patients with normal natrium. Comorbidity is therefore an unlikely explanation for the dismal prognosis for patients with hyponatremia.

Cancer may be accompanied by paraneoplastic phenomenons causing hyponatremia and hypercalcaemia, among others (Kacprowicz and Lloyd, 2009). In small cell lung cancer, the syndrome of inappropriate anti-diuretic hormone secretion (SIADH or Schwartz Batter syndrome) is well described and has been associated with a reduced survival (Gandhi and Johnson, 2006). The SIADH is a likely explanation, however, no reports on SIADH-disturbances exist in RCC. In this study, we were not able to assess whether hyponatremia was associated with SIADH, but the potential association should be examined in future prospective studies.

Other explanations for low sodium may be poor function of the adrenal glands (Yokoyama and Tanaka, 2005; Bahrami *et al*, 2009). However, in our material, there was no statistical correlation between low serum sodium and neprectomy, adrenalectomy, or adrenal metastasis. Another reason may be a renal dysfunction in the exchange mechanism of sodium in the tubules. Taken together, these potential mechanisms remain speculative and further studies are needed to rule out the mechanisms behind low serum sodium.

The serum sodium value used for prognostication was based on a single laboratory value measured before initiation of treatment in all patients. For the electronic devise measuring the level of serum sodium, a high specificity and sensitivity has been shown (Palmer *et al*, 1995; Redondo *et al*, 2003). Moreover, the assessment is easy, cheap, reliable, and reproducible.

Limitations of this study are the relative low sample size in both cohorts and the retrospective evaluation of cohort A. We did not analyse the day-to-day variation in serum sodium. No complete data on concomitant medication were available and therefore, we have been unable to correct for use of diuretics.

In conclusion, hyponatremia is a new independent prognostic and predictive factor in patients with mRCC. Further investigations are needed to examine the mechanisms behind hyponatremia and the association with cancer.

## Figures and Tables

**Table 1:** Baseline characteristics (*N*=120), cohort A

	***N* (%)**
Median age, years (range)	58.3 (29–73)
Male	82 (68)
	
*Performance status*	
0	24 (20)
1	67 (56)
2	28 (23)
3	1 (1)
	
*Previous therapy:*	
Nephrectomy	59 (49)
	
*Number of disease sites*	
1	17 (14)
2	35 (29)
3	35 (29)
4 or more	33 (28)
	
*Most common sites of disease*	
Lymph node	80 (67)
Lung/pleura	74 (62)
Primary kidney tumor	61 (51)
Liver	36 (30)
Bone	30 (25)
Local recurrence kidney bed	18 (15)
Adrenal	14 (12)
Other	15 (13)
	
*Histology*	
Clear cell carcinoma	111 (93)
Papillary	4 (3)
Chromofobe	2 (2)
Unclassified	3 (3)
	
*MSKCC prognostic criteria:* a	
Favourable	15 (13)
Intermediate	65 (57)
Poor	35 (30)

a Memorial Sloan Kettering Cancer Center (Motzer *et al*, 1999).

**Table 2a:** Correlation between baseline factors and serum sodium, cohort A

	**Serum sodium**		
	**Low (*N*=24)**	**Normal (*N*=96)**	***P***
*MSKCC (N=115)*	N (%)	N (%)	0.001
Favourable (*N=*15)	0 (0)	15 (16)	
Intermediate (*N=*65)	8 (36)	57 (61)	
Poor (*N=*35)	14 (64)	21 (23)	
			
*Weight loss (N=119)*			0.001
⩽10% (*N=*95)	13 (54)	82 (86)	
>10% (*N=*24)	11 (46)	13 (14)	
			
*Performance status (N=120)*			0.000
0.1 (*N=*91)	8 (33)	83 (86)	
2+ (*N=*29)	16 (67)	13 (14)	
			
*Number of sites (N=120)*			0.020
1.2 (*N=*52)	5 (21)	47 (49)	
3+ (*N=*68)	19 (79)	49 (51)	
			
*Primary tumor in place (N=120)*			0.039
No (*N=*59)	7 (29)	52 (54)	
Yes (*N=*61)	17 (71)	44 (46)	
			
*Leukocytes (N=120)*			0.029
Normal (*N=*92)	14 (58)	78 (81)	
High (*N=*28)	10 (42)	18 (19)	
			
*Albumin (N=119)*			0.000
Normal (*N=*68)	2 (8)	66 (69)	
Low (*N=*51)	22 (92)	29 (31)	
			
*Alkalic phosphatase (N=118)*			0.004
Normal (*N=*71)	7 (29)	64 (67)	
High (*N=*47)	15 (63)	32 (33)	
			
*Haemoglobin (N=120)*			0.000
Normal (*N=*56)	2 (8)	54 (56)	
Low (*N=*64)	22 (92)	42 (44)	

**Table 2b:** Correlation between treatment factors and serum sodium, cohort A

	**Serum sodium**		
	**Low (*N=*24)**	**Normal (*N=*96)**	***P***
*Response (N=120)*	*N* (%)	*N* (%)	0.003
CR + PR (*N=*17)	2 (8)	15 (16)	
SD (*N=*36)	2 (8)	34 (35)	
PD (*N=*47)	11 (46)	36 (38)	
NE (*N=*20)	9 (38)	11 (11)	
			
*Duration of IL-2 (N=120)*			0.001
⩽3 months (*N=*65)	20 (83)	45 (47)	
>3 months (*N=*55)	4 (17)	51 (53)	

**Table 3:** Overall survival, cohort A, univariate analyses (*N=*120)

**Risk factors**	**Categories**	**Median survival (months)**	**Hazard ratio**	**95 % CI**	***P***
Performance status	2+/0.1	7.2/18.3	1.95	1.25–3.04	0.003
Metastasis-free interval	<1/>1 year	12.5/24.4	1.75	1.11–2.77	0.016
Number of sites	3+/1.2	11.4/23.3	1.71	1.15–2.54	0.008
Primary tumor	+/−	12.7/18.2	1.52	1.03–2.25	0.034
Bone metastases	+/−	10.2/18.2	1.96	1.27–3.02	0.002
Serum sodium	Low/normal	5.5/18.6	2.43	1.51–3.92	0.000
Neutrophils	>7/<7	9.4/20.2	1.66	1.05–2.61	0.030
Albumin	Low/normal	8.9/21.8	1.88	1.27–2.78	0.002
LDH	>1.5/<1.5ULN	10.0/20.2	1.77	1.12–2.80	0.014

**Table 4a:** Overall survival, cohort A, multivariate analysis (*N=*115)

**Risk factors**	**Categories**	**Hazard ratio**	**95% CI**	***P***
Serum sodium	Low/normal	1.86	1.12–3.11	0.017
Number of sites	3+/1.2	1.72	1.11–2.67	0.015
Neutrophils	>7/<7	1.66	1.03–2.65	0.036
LDH	>1.5/<1.5ULN	2.12	1.33–3.40	0.002
Bone metastases	+/−	1.60	1.00–2.56	0.050

**Table 4b:** Overall survival, cohort B, multivariate analysis (*N=*114)

**Risk factors**	**Categories**	**Hazard ratio**	**95 % CI**	***P***
Serum sodium	Low/normal	2.98	1.54–5.77	0.001
Haemoglobin	Low/normal	1.69	1.08–2.63	0.021
Neutrophils	>7/<7	1.74	1.04–2.93	0.036
LDH	>1.5/<1.5 ULN	4.11	2.01–8.44	0.001
Bone metastases	+/−	1.77	1.16–2.70	0.008
